# Coronary Compliance Modification by Intravascular Lithotripsy: New Predictor of Stent Expansion in Calcified Coronary Lesions

**DOI:** 10.1016/j.jscai.2025.102635

**Published:** 2025-05-01

**Authors:** Federico Oliveri, Martijn J.H. Van Oort, Ibtihal Al Amri, Brian O. Bingen, Bimmer E. Claessen, Aukelien C. Dimitriu-Leen, Joelle Kefer, Hany Girgis, Tessel Vossenberg, Frank Van der Kley, J. Wouter Jukema, Josè M. Montero-Cabezas

**Affiliations:** a Department of Cardiology, Leiden University Medical Center, Leiden, the Netherlands; b Department of Cardiology, Amsterdam University Medical Center, Amsterdam, the Netherlands; c Department of Cardiology, Radboud University Medical Center, Nijmegen, the Netherlands; d Department of Cardiology, Saint-Luc Bruxelles, Brussels, Belgium; e Department of Cardiology, Jeroen Bosch Ziekenhuis, Den-Bosch, the Netherlands; f Department of Cardiology, Medisch Centrum Leeuwarden, Leeuwarden, the Netherlands; g Netherlands Heart Institute, Utrecht, the Netherlands

**Keywords:** coronary artery compliance, intracoronary lithotripsy, vascular compliance

## Abstract

**Background:**

Intravascular lithotripsy **(**IVL) has been demonstrated to be effective in treating balloon-crossable calcified coronary lesions by inducing calcium fractures and facilitating stent expansion (SE), theoretically by improving coronary artery compliance (CACom). Direct evidence of this theory has not yet been provided.

**Methods:**

From the BENELUX-IVL prospective registry (NCT06577038) enrolling patients with calcified coronary artery lesions treated with IVL, intravascular ultrasound–guided cases were selected. CACom was calculated as the systo-diastolic change in the luminal area (ΔA), measured using intravascular ultrasound, relative to the corresponding change in aortic pressure (ΔP). Measurements were taken directly before (pre-CACom) and after (post-CACom) IVL therapy at the most calcified segment, where IVL pulses were administered. The primary end point was CACom modification (ΔCACom), defined as post-CACom – pre-CACom, with a correlation analysis between ΔCACom and new fractures as a key exploratory aim. Secondary analysis included assessing whether ΔCACom could predict SE at the minimum stent area (MSA) and the eccentricity index.

**Results:**

Coronary artery compliance significantly improved after IVL therapy (median ΔCACom 0.33 [0.19-0.70] mm^2^/mm Hg; *P* < .01). Lesions showing new calcium fractures presented significantly greater ΔCACom compared to those without. ΔCACom and new calcium fractures were significantly correlated (R = 0.466; *P* < .01). In univariate analysis, ΔCACom was found to be a significant predictor for SE at MSA (*P* < .01), MSA (*P* = .015), and SE >80% (*P* = .025), but not eccentricity index (*P* = .157). At multivariate analysis, ΔCACom was an independent predictor of SE (R = 0.420; *P* = .044) and SE >80% at MSA (OR, 6.58 [1.24-34.90]; *P* = .043).

**Conclusions:**

In heavily calcified coronary lesions treated with IVL, ΔCACom is an independent predictor of SE.

## Introduction

Despite technological and knowledge advancements, percutaneous coronary intervention (PCI) for heavily calcified coronary lesions continues to present significant challenges. The severity of coronary artery calcification is a well-known predictor of stent underexpansion, which is associated with in-stent restenosis and stent thrombosis.^1^ Current plaque modification techniques must strike a delicate balance: insufficient lesion preparation increases the risk of stent underexpansion, whereas overly aggressive preparation raises the risk of procedural complications, such as vessel perforation and dissection. Indeed, a reliable, real-time marker of “sufficient lesion preparation” (degree of calcific plaque modification ensuring adequate stent expansion [SE]) is essential for optimizing PCI outcomes.

Coronary artery compliance (CACom), which reflects the capacity of the vessel to expand and contract in response to aortic pressure changes during the cardiac cycle, may offer such a solution. CACom can be invasively measured via intravascular ultrasound (IVUS) by assessing dynamic changes in the vessel lumen during systole and diastole.^2^, ^3^, ^4^ Several factors, including atherosclerotic burden, vessel location, and coronary physiology, affect CACom.^4^, ^5^, ^6^ For instance, the left main coronary artery shows greater compliance due to its larger size and proximity to the aorta, whereas high calcific plaque burden drastically reduces CACom, making it a potential indicator of lesion severity.^4^

Intravascular lithotripsy (IVL) has broadly demonstrated to be effective in treating heavily calcified coronary lesions by inducing calcium fractures, which theoretically might improve CACom and facilitate SE. However, direct evidence has not yet been provided. In this regard, we have already demonstrated that there is a significant improvement in CACom after IVL therapy of heavily calcified coronary lesions.^7^ However, it remains unclear whether this improvement in CACom directly correlates with improved PCI outcomes. Indeed, we hypothesize that modifying CACom could serve as a reliable surrogate marker of optimal lesion preparation, ultimately guiding successful PCI and offering a novel approach to treating calcified coronary lesions.

## Methods

### Population and data collection

BENELUX-IVL is an international, multicenter, prospective registry (NCT06577038) including all-comers aged >18 years who underwent IVL during PCI. From this registry, patients who underwent intracoronary imaging were reviewed. Inclusion criteria required that patients had undergone IVUS-guided PCI for the treatment of heavily calcified coronary lesions. IVUS had to be performed immediately before and after IVL, as well as after stent deployment. At least 2 cardiac cycles from the most calcified segment, where IVL pulses were administered, needed to be available to accurately calculate CACom. Exclusion criteria were low-quality images, in-stent lesions, lesions requiring up-front rotational/orbital atherectomy, and lesions treated with drug-eluting balloons. Patients unable to provide informed consent were also excluded from the study. For all the IVL procedures, the Shockwave Intravascular Lithotripsy Coronary System (Shockwave Medical) was employed. Technical decisions related to IVL, including timing, balloon size, number of pulses, and maximum pressure, as well as the use of high-pressure predilation and postdilation, were made at the operating physician's discretion. These decisions were systematically recorded in the procedural documentation. We collected demographic, procedural, clinical, and follow-up data from the hospital's electronic health records. IVUS imaging data were analyzed centrally in the core laboratory at the Leiden University Medical Center. The examination of clinically gathered data received approval from the local ethics committees of each participating institution.

### Definitions and imaging analysis

**Figure 1:**
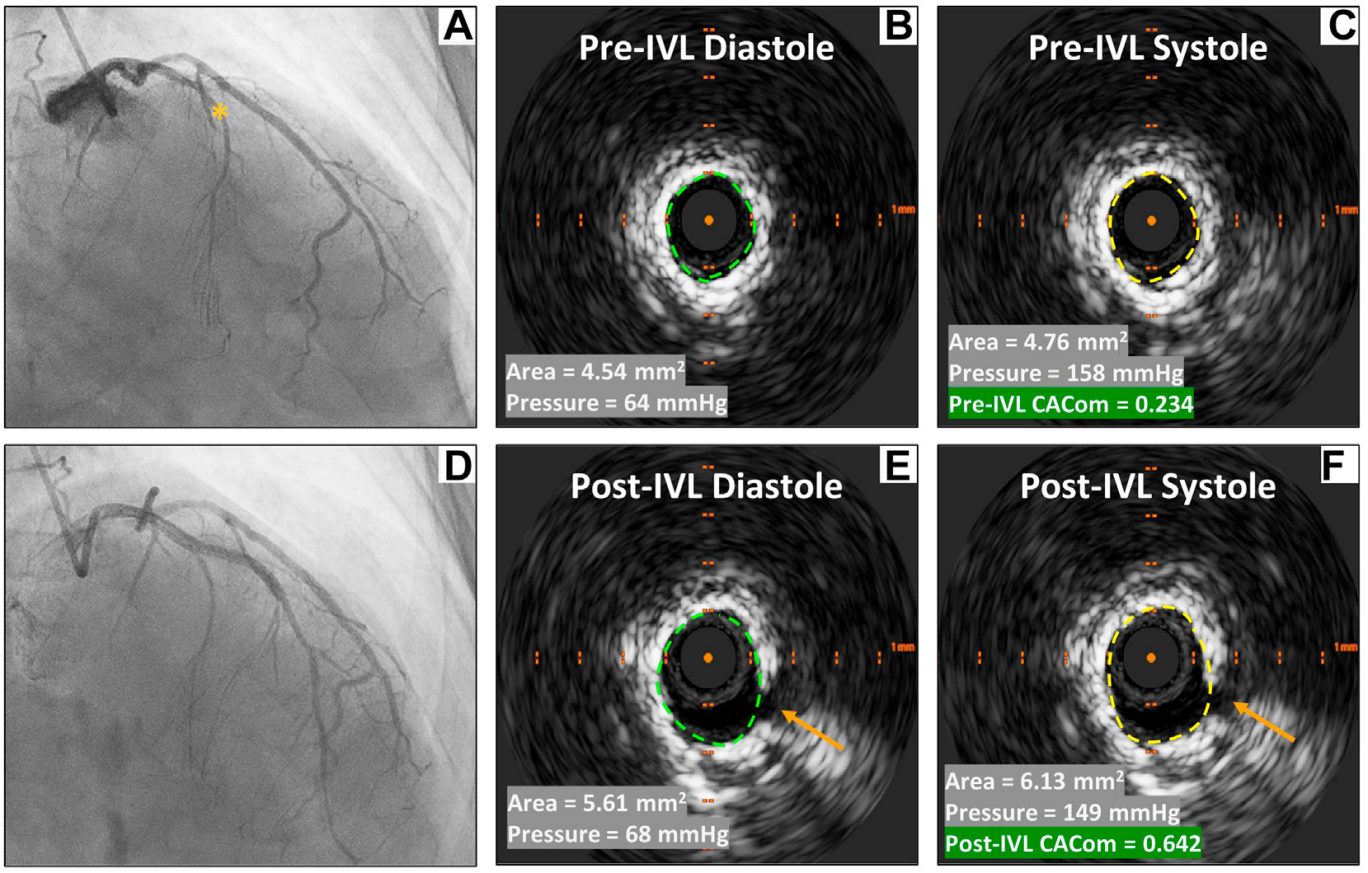
**Coronary angiography and intravascular ultrasound images in a patient with anterior ST-elevation myocardial infarction.** Occlusion in the mid–left anterior descending artery (asterisk) was assessed (**A**). Intravascular ultrasound revealed a long, 360° calcification in the culprit vessel. The luminal area was measured in diastole (**B**) and systole (**C**) before intravascular lithotripsy (IVL) pulse administration, while aortic pressure was recorded simultaneously. Pre-IVL coronary artery compliance was then calculated. A long, 4.0 × 34 mm drug-eluting stent was deployed (**D**). Before stenting, the luminal area and aortic pressure were measured again, both in diastole (**E**) and systole (**F**). Calcium fracture was assessed (arrow). Post-IVL coronary artery compliance was calculated.

Although the majority of coronary blood flow takes place during diastole, expansion of epicardial vessels mainly occurs during systole.^1^^,^^3^ Modification of the luminal area (ΔA) was invasively evaluated using IVUS, with the smallest luminal area identified at peak diastole and the largest luminal area determined at peak systole, all within a single cardiac cycle.^1^^,^^3^ All IVUS images were acquired manually, with stationary recordings taken at the most calcified segments for at least 2 cardiac cycles. The highest-quality cardiac cycle was then selected, and the lumen area at the most calcified segment was measured 5 times during both systole and diastole, totaling 10 measurements. Extreme values were excluded, and the mean lumen areas were calculated separately for systole and diastole, based on the remaining 3 measurements for each phase. Modification in aortic pressure (ΔP), measured at the tip of the guiding catheter, was determined by reviewing the hemodynamic data obtained during IVUS. We excluded cases performed with an 8Fr guiding catheter to avoid pressure measurement distortion. CACom was therefore obtained as follows: ΔA/ΔP × 100.^1^ CACom measurements were conducted both just before and after IVL in the most calcified segment covered by the IVL balloon (Figure 1). The ΔCACom was calculated by subtracting the CACom obtained before IVL (pre-CACom) from the CACom obtained after IVL (post-CACom). Angiographic and IVUS images were evaluated by 2 independent expert operators.

Severe calcification was assessed in accordance with the most recent European Association of Percutaneous Cardiovascular Interventions consensus. Specifically, a calcium score of ≥2 on IVUS was required, defined by meeting at least 2 of the following criteria: 360° calcification, calcium extending over >270° and measuring ≥5 mm in length, the detection of a calcified nodule, or a vessel diameter less than 3.5 mm.^8^ The Philips IVUS system was used in conjunction with the Eagle Eye Platinum ST catheter (Philips).

Analysis of IVUS was performed using QCU-CMS 4.69 (Leiden University Medical Center). The primary IVUS parameters were evaluated based on the European Association of Percutaneous Cardiovascular Interventions consensus on the clinical use of intracoronary imaging.^9^ The eccentricity index (EI) was calculated by subtracting the minimum lesion diameter from the maximum lesion diameter at the minimum stent area (MSA) and then dividing this difference by the maximum lesion diameter. SE at the MSA was assessed by dividing the MSA by the reference vessel area. Additionally, the presence of new calcium fractures at the sites where IVL pulses were administered was documented. Preexisting calcium fractures detected after high-pressure dilation performed prior to IVL were not included in the count of new calcium fractures.

### Study end points

The primary end point was the modification of CACom (ΔCACom), defined as post-CACom – pre-CACom, following IVL pulse administration, with a correlation analysis between ΔCACom and the presence of new calcium fractures as a key exploratory aim. The secondary end point of this study is to assess whether post-CACom and/or ΔCACom can be independent predictors of SE at the MSA, SE greater than 80%, MSA or EI.

### Statistical analysis

Demographic, procedural, and imaging data were presented for the included patients. Continuous variables are presented as either mean ± SD or median with IQR (25th to 75th percentile), depending on their distribution. Normality was visually assessed by drawing Q-Q plots and checked by the Shapiro-Wilk statistic. Paired continuous variables were evaluated using the paired *t* test for normally distributed data and the Wilcoxon signed-rank test for nonnormally distributed data. Unpaired continuous variables were assessed with the unpaired *t* test for normally distributed and with the Mann-Whitney U test for nonnormal distributions. Categorical variables were expressed as frequencies and percentages and analyzed using the χ^2^ or Fisher exact test, when appropriate.

A preliminary bivariate correlation analysis was conducted to explore relationships between variables. For continuous variables, Pearson's correlation coefficient was used for normally distributed variables and Spearman's rank correlation for nonnormally distributed variables or if outliers were present. Subsequently, a univariate linear regression analysis was performed to investigate the relationship between potential predictors and continuous dependent variables (SE at MSA, MSA, and EI). For the dichotomous dependent variable (SE > 80%), a univariate binary logistic regression analysis was instead conducted. To identify independent predictors for the considered outcomes (SE, MSA, EI, SE > 80%, and EI), multivariate linear and logistic regression analyses were performed. The multivariate regression analysis was conducted twice (because of new potential predictors): once using ΔCACom and again with post-CACom, if deemed pertinent following the univariate analysis. The models included predictors related to the outcomes (*P* < .20 in univariate analysis) and a priori potential confounders (age, sex, stent maximum diameter, calcium arc at the minimum lumen area, post-IVL balloon maximum pressure, and post-IVL balloon maximum diameter). Multicollinearity was assessed using the variance inflation factor, with a threshold of <10 indicating that multicollinearity was not present. Box plots, scatter plots, and coefficient plots were generated to visually present the analysis results. All tests were 2-sided, with a significance level of *P* < .05. Statistical analyses were conducted using SPSS for Windows, version 25.0 (IBM).

## Results

Between May 2019 and February 2024, a total of 455 patients were prospectively included in our registry. Of them, 49 patients (and 49 lesions) matched our inclusion criteria (Tables 1 and 2). The mean age was 74 ± 4.4 years, with nearly one-fourth (22.4%) being female. The left ventricular ejection fraction was 51% ± 5.7%. Stable angina was the most common clinical indication for PCI (32.7%), followed by NSTEMI (22.4%). A history of previous PCI was noted in 20.4% of the patients. The majority of patients (59.2%) were on beta-blockers as part of their antianginal therapy. The median procedural time and administered contrast volume were 90 ± 12 minutes and 200 (143-250) mL, respectively. The radial artery was the preferred access site for PCI in 81.6% of cases. The left anterior descending artery was the most commonly targeted vessel (44.9%), followed by the right coronary artery (42.9%). The mean SYNTAX score was 17.8 ± 4.1. Pre-IVL high-pressure dilation was performed in nearly all lesions (98%). The pre-IVL maximum balloon diameter and inflation pressure were 3.0 (3.0-3.5) mm and 18 (16-20) atm, respectively. Post-IVL high-pressure dilation was performed in all cases (100%). The post-IVL maximum balloon diameter and inflation pressure were 4.0 (3.5-4.0) mm and 22 (20-24) atm, respectively. Significant vessel dissection (with TIMI flow grade I) occurred in 1 case (2.0%), which was successfully treated with the deployment of an additional stent. No further intraprocedural complications were observed.

**Table 1:** Baseline characteristics.

Variable	N = 49
Age, y	74 ± 4.4
Female sex	11 (22.4)
Body mass index, kg/m^2^	23 (22.5-27.1)
Hypertension	33 (67.3)
Left ventricular ejection fraction	51 ± 5.7
Dyslipidemia	22 (44.9)
Smoking history	22 (44.9)
Diabetes mellitus	17 (34.7)
Chronic kidney disease (eGFR <60 mL/min/1.73 m^2^)	15 (30.6)
Previous PCI	10 (20.4)
Previous stroke/transient ischemic attack	7 (14.3)
Clinical presentation	
Stable angina	16
Unstable angina	8
NSTEMI	11
STEMI	6
Others	8
Anti-ischemic medications	
Beta-blockers	29 (59.2)
Nitrates	8 (16.3)

Values are mean ± SD, n (%), or median (IQR).

eGFR, estimated glomerular filtration rate (using the Modification of Diet in Renal Disease formula); NSTEMI, non–ST-elevation myocardial infarction; PCI, percutaneous coronary intervention; STEMI, ST-elevation myocardial infarction.

**Table 2:** Procedural characteristics.

Characteristic	N = 49
Procedural time, min	90 ± 12
Contrast volume, mL	200 (143-250)
Access	
Radial	40 (81.6)
Femoral	9 (18.4)
Vessel	
Left main artery	3 (6.1%)
Left anterior descending artery	22 (44.9%)
Circumflex artery	3 (6.1%)
Right coronary artery	21 (42.9%)
SYNTAX score	17.8 ± 4.1
Bifurcation	7 (14.3)
Ostial	12 (24.4)
Chronic total occlusion	8 (16.3)
Need for inotropes	2 (4.1)
Need for mechanical support	2 (4.1)
Intraaortic balloon pump	1 (2.0)
Impella	1 (2.0)
VA-ECMO	0 (0)
Pre-IVL high-pressure dilatation	42 (85.7)
Pre-IVL largest balloon, mm	3.0 (3.0-3.5)
Pre-IVL maximum pressure dilatation, atm	18 (17-20)
IVL pulses delivered	
≤40	2 (4.1%)
41-79	17 (34.7%)
80	29 (59.2%)
>80	7 (14.3%)
Maximum diameter IVL balloon, mm	3.5 (3.5-4.0)
Post-IVL high-pressure dilatation	49 (100%)
Post-IVL largest balloon, mm	4.0 (3.5-4.0)
Post-IVL maximum pressure dilatation, atm	22 (20-24)
Total stent length, mm	38 (26-60)
Stent maximum diameter, mm	4.0 (3.5-4.0)
Procedural complication	1 (2.0)
Severe coronary dissection (D-E-F)	1 (2.0)
Abrupt vessel closure	0
Perforation	0
Tamponade	0
Equipment loss	0
Life-threatening arrhythmias	0
Myocardial infarction	0

Values are mean ± SD, median (IQR), or n (%).

IVL, intravascular lithotripsy; VA-ECMO, venous-arterial extracorporeal membrane oxygenator.

### Procedural characteristics

**Figure 2:**
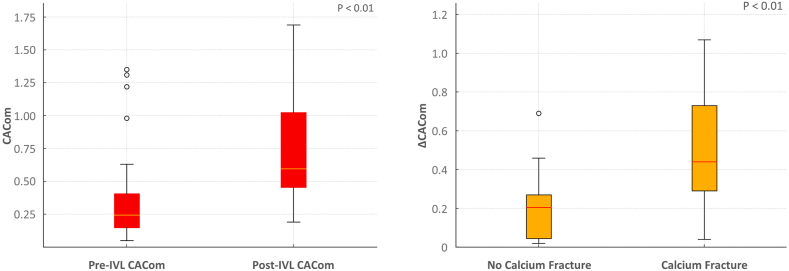
**Box plots showing the median coronary artery compliance (CACom) before and after intravascular lithotripsy (IVL) therapy (left panel).** A significant increase in median CACom was observed. Lesions showing evidence of new calcium fractures exhibited a significantly greater increase in CACom compared to those without fractures (right panel).

Table 3 presents the IVUS characteristics and procedural outcomes. The mean reference vessel diameter was 4.20 ± 0.07 mm. The pre-IVL minimum lumen area and area of stenosis were 3.72 ± 0.21 mm^2^ and 72.5% ± 1.6%, respectively. The median maximum calcium angle was 360° (305°-360°). Post-IVL, the MSA was 10.19 ± 0.34 mm^2^, and SE at the MSA was 78.6% ± 1.6%. An MSA >5.5 mm^2^ (or >8 mm^2^ for the left main artery) was achieved in 98% of cases. The stent EI at the MSA was 0.18 ± 0.02. The median pre-IVL and post-IVL CACom were 0.25 (0.15-0.46) mm^2^/mm Hg and 0.61 (0.46-1.03) mm^2^/mm Hg, respectively, with a ΔCACom of 0.33 (0.19-0.70) mm^2^/mm Hg (*P* < .01). Calcium fractures were observed in 75.5% of cases. Target lesions with new calcium fractures observed on IVUS showed a higher compliance modification (ΔCACom) than those without (Figure 2). A moderate correlation between ΔCACom and new calcium fractures was assessed (R = 0.466; *P* value < .01). Device success, defined as procedural success with residual stenosis of less than 30%, was achieved in 98.0% of cases. In-hospital major adverse cardiac events occurred in 1 patient (2.0%), resulting in a cardiac death.

**Table 3:** IVUS characteristics and procedural outcomes.

Characteristic	N = 49
Reference vessel diameter, mm	4.20 ± 0.07
Reference vessel area, mm^2^	13.51 ± 0.44
Pre minimum lumen diameter, mm	1.88 (1.70-2.20)
Pre minimum lumen area, mm^2^	3.72 ± 0.21
Pre area stenosis, %	72.5 ± 1.6
Max persistent Ca^2+^ angle, °	360 (305-360)
Post minimum stent area, mm^2^	10.19 ± 0.34
Post minimum stent area >5.5 (or 8 mm^2^ for LM)	48 (98.0)
Poststent expansion at MSA, mm^2^	78.6 ± 1.6
Stent expansion >80%	22 (44.9)
Post eccentricity index at MSA	0.18 ± 0.02
Calcium fracture	37 (75.5)
Pre-CACom, mm^2^/mm Hg	0.25 (0.15-0.46)
Post-CACom, mm^2^/mm Hg	0.61 (0.46-1.03)
ΔCACom, mm^2^/mm Hg	0.33 (0.19-0.70)
Device success	48 (98.0)
Procedural success <30%	48 (98.0)
In hospital	
Major adverse cardiac events	1 (2.0)
Cardiac death	1 (2.0)
Target vessel revascularization	0 (0)

Values are mean ± SD, median (IQR), or n (%).

CACom, coronary artery compliance; IVUS, intravascular ultrasound; LM, left main artery; MSA, minimal stent area.

### Univariate regression analysis

Table 4 lists the potential predictors identified through univariate regression analysis for the studied end points. ΔCACom was found to be a significant predictor for SE at MSA (*P* < .01), MSA (*P* = .015), and SE >80% (*P* = .025). There was no significant correlation between ΔCACom and EI (*P* = .157).

**Table 4:** Predictor of stent success—Univariate regression.

Predictors	SE at MSA	SE >80%	MSA	EI
Age	0.084	–	–	–
Body mass index	0.095	–	0.149	–
Chronic total occlusion	–	–	0.103	–
Contrast volume	–	–	0.024	–
Dyslipidemia	-	0.055	–	0.047
Fluoroscopic time	–	–	–	0.017
Glomerular filtration rate	–	–	–	0.067
Hypertension	0.154	–	–	–
IVL maximum balloon diameter	0.166	–	<0.01	–
Left anterior descending artery	0.083	–	–	–
Left main artery	–	–	–	0.113
Lesion length >20 mm	–	–	–	0.039
New stent maximum diameter	<0.01	–	<0.01	–
New stent maximum length	–	–	–	<0.01
Number of IVL balloon inflations	–	–	–	0.029
Number of IVL pulses delivered	–	–	0.106	0.185
Post-IVL maximum pressure dilatation	–	–	–	0.017
Sex	–	–	0.107	0.094
Smoking	0.103	0.048	0.144	–
ΔCACom	<0.01	0.025	0.015	0.157
Post-IVL CACom	–	–	0.029	–

Numbers represent *P* values obtained in the univariate analysis. An en dash (–) was inserted where the *P* value was >.20.

CACom, coronary artery compliance; EI, eccentricity index; IVL, intravascular lithotripsy; MSA, minimum stent area; SE, stent expansion.

**Supplementary Figure S1:**
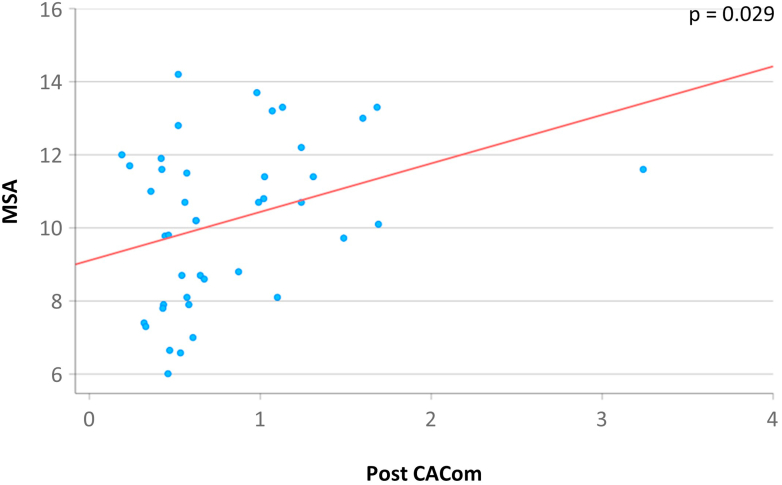


Post-CACom was significantly associated with MSA only (*P* = .029) (Supplemental Figure S1), but not with SE at MSA or EI.

### Multivariate regression analysis

**Figure 3:**
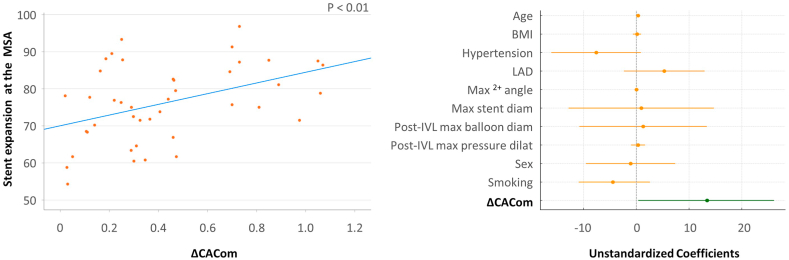
**Scatter plot showing a positive correlation between ΔCACom and stent expansion at the minimum stent area (MSA) (left panel).** In the multivariate analysis, ΔCACom was the only independent predictor of stent expansion at the MSA (right panel). BMI, body mass index; CACom, coronary artery compliance; IVL, intravascular lithotripsy; LAD, left anterior descending artery.

**Figure 4:**
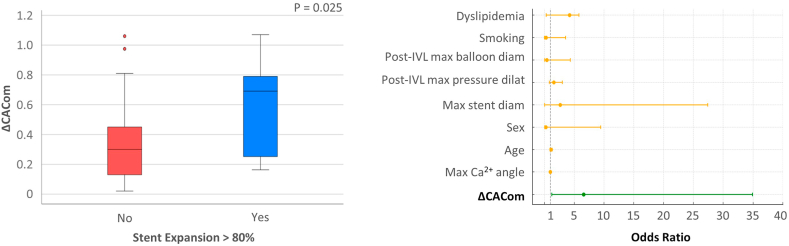
**In the univariate analysis, ΔCACom was found to be a significant predictor for stent expansion (SE) >80% at minimum stent area.** Percutaneous coronary intervention with a final SE >80% presented a greater ΔCACom (left panel). In the multivariate analysis, the coefficient plot shows ΔCACom as the only independent predictor of SE at the minimum stent area (right panel). CACom, coronary artery compliance; IVL, intravascular lithotripsy.

**Supplementary Figure S2:**
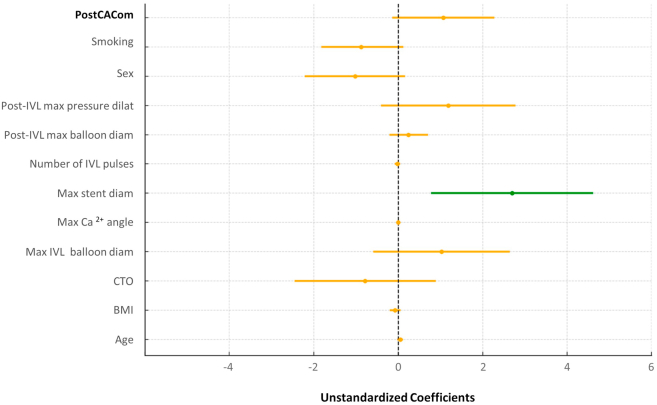


**Supplementary Figure S3:**
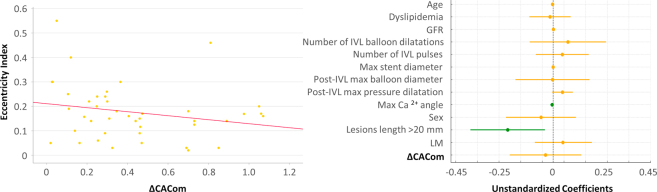


Table 5 presents the predictors for the end points considered in our analysis. ΔCACom was the only predictor of SE at MSA (R = 0.420; *P* = .044) and SE >80% at MSA (OR, 6.58; 95% CI, 1.24-34.90; *P* = .043) (Figures 3 and 4). The stent's maximum diameter was significantly correlated with MSA (R = 0.417; *P* = .013), whereas ΔCACom was not (R = 0.207; *P* = .117) (Supplemental Figure S2). Independent predictors of EI were post-IVL maximum pressure dilatation (R = –0.425; *P* = .033) and IVL pulses delivered (R = –0.494; *P* = .045), whereas ΔCACom was not a significant predictor (R = –0.09; *P* = .654) (Supplemental Figure S3).

**Table 5:** Predictors of stent success—Multivariate regression.

Stent expansion at minimum stent area		
Predictors	R	*P* value
Age	.339	.07
Body mass index	.074	.69
Hypertension	(–) .356	.08
Left anterior descending artery	.268	.16
Max Ca^2+^ angle	.221	.21
New stent maximum diameter	.034	.89
Post-IVL largest balloon diameter	.061	.83
Post-IVL maximum pressure dilatation	.097	.59
Sex	(–) .043	.80
Smoking	(–) .211	.22
ΔCACom	.420	.044

Stent expansion >80%		

Predictors	OR (95% CI)	*P* value

Age	1.11 (0.94-1.31)	.217
Dyslipidemia	4.21 (0.31-5.80)	.283
Max Ca^2+^ angle	0.99 (0.97-1.01)	.562
New stent maximum diameter	2.66 (0.03-27.4)	.679
Post-IVL largest balloon diameter	0.39 (0.03-4.35)	.178
Post-IVL maximum pressure dilatation	1.59 (0.84-3.03)	.156
Sex	0.25 (0.06-9.44)	.452
Smoking	0.27 (0.02-3.56)	.322
ΔCACom	6.58 (1.24-34.90)	.043

Minimum stent area		

Predictors	R	*P* value

Age	.168	.153
Body mass index	(–) .179	.163
Chronic total occlusion	(–) .181	.144
IVL maximum balloon diameter	.205	.133
Max Ca^2+^ angle	(–) .115	.323
New stent maximum diameter	0.417	.013
Number of IVL pulses delivered	(–) .121	.348
Post-IVL largest balloon diameter	.176	.339
Post-IVL maximum pressure dilatation	.055	.695
Sex	(–) .200	.09
Smoking	(–) .198	.104
ΔCACom	.207	.117

Eccentricity index		

Predictors	R	*P* value

Age	(–) .217	.337
Dyslipidemia	(–) .06	.746
Glomerular filtration rate	.202	.393
Left main	.204	.425
Lesion length >20 mm	.136	.470
Max Ca^2+^ angle	(–) .025	.907
Stent maximum diameter	(–) .03	.975
Number of IVL balloon inflations	.556	.066
Number of IVL pulses delivered	(–).494	.045
Post-IVL largest balloon diameter	.216	.470
Post-IVL maximum pressure dilatation	(–) .425	.033
Sex	.154	.468
ΔCACom	(–).09	.654

CACom, coronary artery compliance; EI, eccentricity index; IVL, intravascular lithotripsy; MSA, minimum stent area; OR, odds ratio; SE, stent expansion.

**Supplementary Figure S4:**
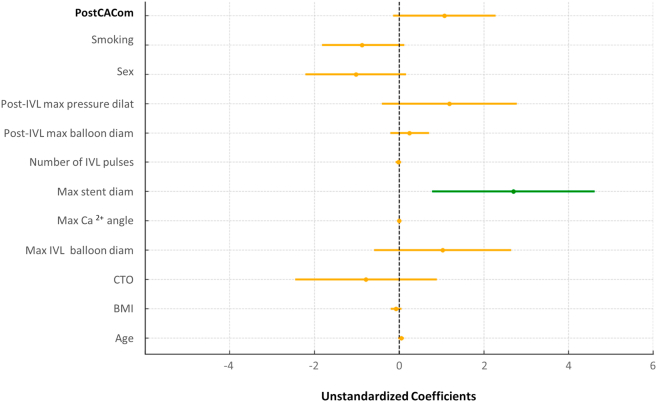


Post-CACom was not significantly associated with MSA in the multivariate analysis (R = 0.218; *P* value = .08) (Supplemental Figure S4).

## Discussion

The current study aimed to assess the relation between CACom, calcium fractures, and stent outcomes in patients undergoing PCI for heavily calcified coronary lesions treated with IVL. Our key findings were as follows: (1) significant improvement in CACom following IVL; (2) significant correlation between ΔCACom and the presence of calcium fractures detected by IVUS; (3) ΔCACom, but not post-CACom, was an independent predictor of SE and SE >80% at the MSA (Central Illustration).

**Central Illustration:**
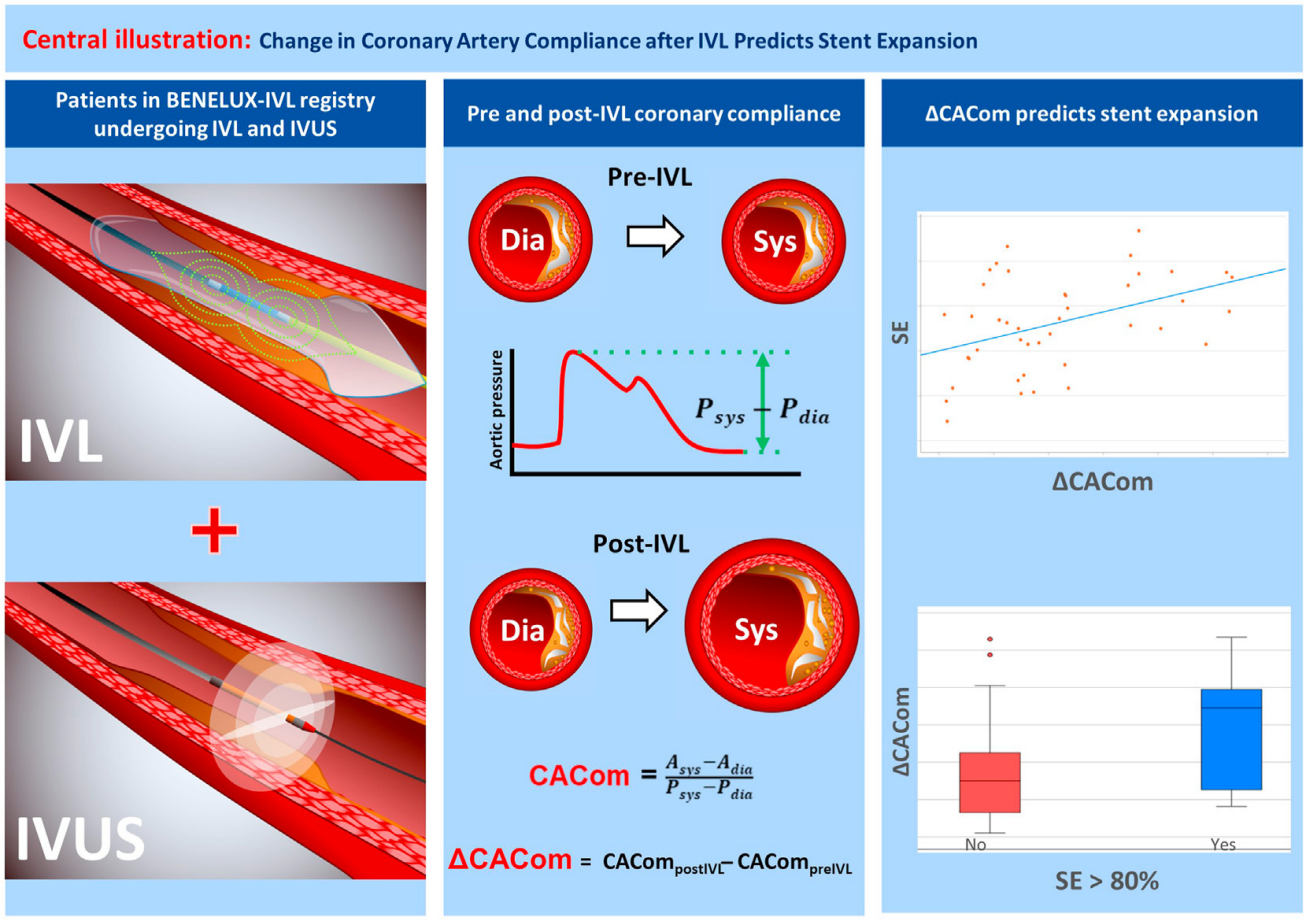
**Change in coronary arterycompliance (ΔCACom) after intravascular lithotripsy (IVL) predicts stent expansion.** IVUS, intravascular ultrasound; SE, stent expansion.

Despite advancements in PCI techniques, heavily calcified lesions continue to present significant procedural challenges, often resulting in suboptimal SE.^1^^,^^10^ Pivotal IVL registries have broadly demonstrated the efficacy and safety of IVL in treating balloon-crossable calcified lesions.^11^^,^^12^ Although CACom modification secondary to macroscopic calcium fracture was hypothesized to be a mechanism behind IVL success, direct evidence has not yet been provided. We have already demonstrated that there is a significant improvement in CACom after IVL therapy in heavily calcified coronary lesions.^7^ Our study provides novel insights by showing that lesions with post-IVL calcium fracture exhibited greater ΔCACom compared to those without fractures, underscoring the role of calcium fracture in enhancing CACom.

The severity of coronary artery calcification is a known predictor of stent underexpansion, which is associated with adverse outcomes such as in-stent restenosis and stent thrombosis.^1^, ^2^, ^3^, ^4^, ^5^, ^6^, ^7^, ^8^, ^9^, ^10^ Achieving SE >80% is an important procedural goal to mitigate these risks.^10^^,^^11^ However, reaching this target in heavily calcified lesions typically requires aggressive lesion preparation, increasing the likelihood of complications such as vessel perforation or dissection. Our study identified ΔCACom as an independent predictor of SE >80%, highlighting its potential as a real-time, IVUS-based parameter that can be assessed during PCI. Compliance modification provides dynamic feedback on lesion preparation. By improving CACom, operators can be more confident in achieving sufficient SE without resorting to overly aggressive postdilation, which potentially might carry additional procedural risks (eg, coronary dissection or perforation). Indeed, ΔCACom provides live feedback during PCI, which could lead to improved procedural safety and stent success in cases involving complex calcified lesions.

The lack of correlation between post-CACom and considered stent parameters, except for borderline significance with MSA, is consistent with the physiology of calcified lesions. Calcification within coronary plaques has been shown to reduce CACom by more than 4-fold compared to the same vessel segment in the absence of plaque.^4^ As a result, the absolute CACom values in such cases remain low, even after IVL treatment. Post-CACom captures a “snapshot” of the vessel's compliance at a single time point after the intervention, reflecting the vessel's state postintervention. In cases of severe calcification, post-CACom might remain low, even if there was a significant relative improvement in compliance (captured by ΔCACom). On the other hand, ΔCACom, which measures the relative change in compliance from before to after IVL, might provide a better representation of the vessel's response to the lesion preparation. Even if the final post-CACom remains low, a significant improvement in compliance (as indicated by ΔCACom) can reflect successful plaque modification, contributing to a better correlation with SE.

The EI is another common stent parameter evaluated by intracoronary imaging. However, its role as a predictor of stent success is less established compared to others.^13^ Unlike SE, EI was not significantly correlated with ΔCACom. This finding can be explained by the fact that compliance modifications induced by IVL are not always uniform across the vessel wall. As a result, some areas of the vessel wall may become more compliant, whereas others remain stiffer, contributing to eccentric SE. Additionally, fibrotic plaques, which are less responsive to IVL, and the intrinsic geometry of coronary vessels may further contribute to eccentricity.

Thus, we believe that ΔCACom may offer real-time feedback on “sufficient lesion preparation,” enabling operators to optimize stent deployment while minimizing procedural risks.

### Limitations

Several limitations of this study must be acknowledged. First, although patients were prospectively enrolled, the small sample size and the retrospective nature of this analysis introduce potential biases. Second, invasive pressure measurements were taken at the catheter tip, which may not perfectly reflect intracoronary pressure at the lesion site, potentially affecting CACom calculations. Third, IVUS might underestimate the real incidence of calcium fracture. Fourth, medications affecting vascular tone, such as nitroglycerin, may have influenced compliance measurements. However, prior data suggest that nitroglycerin does not significantly alter dynamic vessel dimensions, especially in calcified segments.^9^ Fifth, the ΔCACom threshold above which “sufficient lesion preparation” has been reached, is still to be determined. Finally, although IVL significantly improved CACom in this study, other lesion preparation techniques, such as noncompliant balloons or atherectomy, may also modify compliance, and further studies are necessary to evaluate the optimal approach to lesion preparation.

## Conclusion

Modification of CACom is a robust predictor of SE in patients undergoing PCI for heavily calcified coronary lesions treated with IVL. The ability to assess ΔCACom in real-time during PCI offers a valuable tool for optimizing lesion preparation and reducing the need for aggressive postdilation.

## Footnotes

To access the supplementary material accompanying this article, visit the online version of the *Journal of the Society for Cardiovascular Angiography & Interventions* at 10.1016/j.jscai.2025.102635.
